# Inhibition of Rat Muscle and Liver Phosphofructokinases by High Doses of Ethanol

**DOI:** 10.1155/2013/495135

**Published:** 2013-11-13

**Authors:** Sergey Vladimirovich Lelevich, Vladislav Victorovich Khrustalev, Eugene Victorovich Barkovsky

**Affiliations:** ^1^Department of Clinical Laboratory Diagnostics, Allergology and Immunology, Grodno State Medical University, Gorkogo 80, 230009 Grodno, Belarus; ^2^Department of General Chemistry, Belarusian State Medical University, Dzerzinskogo 83, 220116 Minsk, Belarus

## Abstract

Activities of both rat muscle and liver phosphofructokinases are significantly inhibited after a single ethanol intake in the dose of 2.5 g per kg of body weight. This inhibitory effect is indirect, since ethanol in concentration (50 mM) close to that established after 2.5 g per kg of body weight intake cannot decrease their activities *in vitro*. Inhibition of liver phosphofructokinase activity after the 5.0 g per kg ethanol intake may be direct, since liver phosphofructokinase activity decreases *in vitro* when ethanol is added to supernatants of rat liver tissue in 100 mM concentration. According to the results of molecular docking, ethanol at high concentrations can be bound by adenine-binding pocket of the allosteric ADP-binding site of liver phosphofructokinase (Asp543, Phe308, Phe538, and Phe671) and its activation by ADP can be blocked by C_2_H_5_OH molecule. Direct inhibition of muscle phosphofructokinase activity, probably due to the binding of ethanol to the similar ADP-binding site, is possible when the concentration of ethanol (500 mM) is much higher than the level which can be established in living cells. So, inhibition of muscle phosphofructokinase activity after a single 5.0 g per kg intake is indirect and probably linked with the inhibition of the enzyme by elevated citrate and phosphoenolpyruvate levels.

## 1. Introduction

Phosphofructokinase catalyzes phosphorylation of fructose-6-phosphate to fructose-1,6-bisphosphate. This reaction is a key regulatory step in the glycolysis [1]. Phosphofructokinase activity is regulated through allosteric inhibition and activation. High ATP to ADP ratio inhibits phosphofructokinase and glycolysis as well [1]. Indeed, ADP (the product of the reaction) is an allosteric activator of the activity of that enzyme [2]. Phosphofructokinase is also activated by AMP and fructose-2,6-bisphosphate and inhibited by phosphoenolpyruvate and citrate [3].

Mammalian phosphofructokinase is a tetramer. There are three genes encoding monomers of phosphofructokinase. They are designated as muscle, liver, and platelet phosphofructokinases. The muscle enzyme is a homotetramer (composed of four identical muscle subunits) [1]. Liver also expresses predominantly homotetramer composed of four liver subunits [1].

Ethanol can be found in 163 entries in the Protein Data Bank (http://www.pdb.org/). It can interact with many proteins being a part of the solvent. Alcohol dehydrogenases bind (and metabolize) ethanol specifically [1]. Ethanol also binds *α*7-nAChRs (nicotinic acetylcholine receptors) as an agonist [4], GABA receptors (gamma-aminobutyric acid receptors) as a positive allosteric modulator [5], NMDA receptor (N-methyl-D-aspartate receptor) as an antagonist [6], and glycine receptor as an agonist [7]. Moreover, ethanol activates G protein-gated inwardly rectifying K^+^ channels (GIRK) [8]. A putative alcohol-binding pocket located in the cytoplasmic domains of GIRK channels has been suggested [8]. That alcohol-binding pocket is thought to be similar to the specific alcohol-binding pocket described for the odorant binding protein LUSH from *Drosophila melanogaster* [8–10].

In our recent work we showed that such glycolytic enzyme as pyruvate kinase (both muscle and liver isoenzymes) can also be directly inhibited by very high (500 mM) doses of ethanol [11]. Taking into account the ability of ethanol to form hydrogen bonds and participate in polar, cation-pi, and hydrophobic interactions with proteins, it is very important to check whether its effect on activity of a certain enzyme is direct or indirect. Direct effect is a consequence of ethanol physical binding by a certain binding pocket of a protein [8]. Indirect effect is not caused by the physical binding of ethanol by a protein of interest. Instead of that, ethanol may act through changing the fluidity of the lipid bilayer [8], through the neural or hormonal regulation, through the binding with other proteins, or even DNA [12], and so forth.

The main purpose of this study was to find out whether ethanol can directly inhibit activities of muscle and liver phosphofructokinases. The answer to this question was positive: high concentrations of ethanol decrease phosphofructokinase activity *in vitro*. The second question to solve was whether direct inhibition of phosphofructokinase by ethanol may have any connections with the effects of acute alcohol intoxication on the activity of that enzyme? According to our results, inhibition of muscle phosphofructokinase activity after the acute alcohol intoxication is not a consequence of its direct inhibition by ethanol. Muscle enzyme can be directly inhibited only by 500 mM ethanol. Direct inhibition of liver phosphofructokinase takes place when the concentration of ethanol reaches 100 mM, so it is possible that after the intake of 5.0 g per kg of ethanol its activity can be inhibited directly. 

Differences in structures of allosteric ADP-binding sites for muscle and liver phosphofructokinases have been discussed. Because of those differences ethanol can directly inhibit liver phosphofructokinase activity at lower concentrations than in the case with its muscle homologue.

## 2. Materials and Methods

Acute alcohol intoxication has been modeled in twenty two rats (males, 180–220 g), seven rats have been used as a control group. All the rats have been fasted for 11 hours before the ethanol (or equivolume 0.9% NaCl) admission via stomach intubation. Seven rats received 25% ethanol in water solution in the dosage of 1.0 g per kg of body weight; seven rats received 25% ethanol in dosage of 2.5 g per kg of body weight; and eight rats received 25% ethanol in dosage of 5.0 g per kg of body weight. Rats were decapitated after 1 hour from the ethanol or saline intake. The doses of ethanol used in this study (1.0, 2.5, and 5.0 g per kg of body weight) are standard for experiments on acute ethanol intoxication consequences [13–15].

The dosage of ethanol equal to 5.0 g per kg of body weight is widely used in studies on the effects of heavy acute alcohol intoxication. According to the different sources that dosage of ethanol delivered via stomach intubation results in 80.43 mM [14, 16] or 108.0 ± 2.3 mM [17] peak blood levels.

The dosage of ethanol equal to 2.5 g per kg of body weight is used for models of acute average dose alcohol intoxication. Peak blood levels after a single 2.5 g per kg ethanol intake vary from 23.9 ± 11.3 mM [18], 39.3 ± 10.0 mM [19], 40.43 mM [14] up to 55.0 ± 7.1 mM [20].

The dosage of ethanol equal to 1.0 g per kg of body weight corresponds to the low dose acute alcohol intoxication. Peak blood alcohol levels after that dose of ethanol vary significantly: 7.2 ± 0.43 mM [13], 11.8 ± 1.4 mM [21], 15.9 ± 2.2 mM [22], and 18.3 ± 1.0 mM [23].

*In vitro* experiment on the direct ethanol effect on phosphofructokinases activities has been performed on supernatants of liver and muscle tissues of six rats (males, 180–220 g). We performed experiments with four final concentrations of ethanol (5, 50, 100, and 500 mM). The lowest concentration of ethanol used in *in vitro* experiment (5 mM) corresponds to the lowest dosage of ethanol (1.0 g per kg of body weight) in *in vivo* experiment [13]. The concentration of 50 mM can be established after the intake of 2.5 g per kg of ethanol [20]. The concentration of 100 mM corresponds to 5.0 g per kg ethanol administration [17]. The highest concentration of ethanol (500 mM) was also used to study the direct effect of ethanol on enzymatic activity, even though that concentration cannot be established *in vivo*.

Liver and muscle tissues have been homogenized in glass homogenizers. Supernatants have been obtained after the centrifugation of homogenates.

Enzymatic activity of phosphofructokinase in supernatants of liver and muscle tissues has been measured by a method suggested by Undervud and Newsholme [24] (in the fructose-bisphosphate aldolase, alpha-glycerophosphate dehydrogenase, and triosephosphate dehydrogenase coupled assay). Incubation mixture contained 70 mM tris-HCl buffer pH = 8.2; 2 mM AMP; 1 mM EDTA; 0.2 mM NADH; 1 mM 2-merkaptoethanol; 0.3 mM KCN; 5 mM MgCl_2_; 50 microgram of fructose-bisphosphate aldolase; 10 microgram of the alpha-glycerophosphate dehydrogenase, and 10 microgram of triosephosphate dehydrogenase. All the probes were incubated for 10 minutes at 25°C. Then, fructose-6-phosphate (final concentration is equal to 1 mM) and ATP (final concentration is equal to 1 mM) were added to the test probes. Fructose-6-phosphate was not added to the control probes. Absorption of control probes was measured at 340 nm on “SOLAR” spectrophotometer. Then, test probes were further incubated for 20 minutes at 20°C. Activities have been calculated from the differences in absorbance of test and control probes at 340 nm. Activities are expressed in nmol of substrate per mg of tissue per minute (nmol/mg/min).

There are two X-ray structures of phosphofructokinase (3O8N and 3O8L) submitted to the Protein Data Bank (http://www.pdb.org/). Both of those PDB files describe rabbit muscle phosphofructokinase [2]. In 3O8N file, there are three molecules of ADP bound to three different sites of a single phosphofructokinase [2]. We used 3O8L file as a template to construct 3D models of human and rat muscle and liver homologous enzymes because in that file there are two ATP molecules and a single ADP molecule bound to the protein [2]. One of the three binding sites was thought to be specific for ADP and not ATP binding [2].

Swiss Model server [25] (http://swissmodel.expasy.org/) has been used for the modeling of rat and human enzymes based on their amino acid sequences from GenBank (NP_001160158; AAH94212.1; NP_002617.3; NP_037322.1).

The Docking Server [26, 27] (http://www.dockingserver.com/) has been used for molecular docking of ethanol, ADP, and ATP to 3D models of phosphofructokinases, as well as for docking of ethanol to the LUSH protein of *Drosophila melanogaster* (1OOF). That server automatically used an area for docking which includes a specific site for ADP binding in case of phosphofructokinases and ethanol-binding pocket in case of LUSH. There were 10 runs for each docking. We calculated average levels of free energies of binding for those 10 runs. Then, we calculated binding constants according to the formula given below

(1) $$\begin{array}{c} K=e^{-\Delta G/RT}. \end{array}$$

In that formula, *K* is a binding constant, Δ*G* is a free energy of binding (in kcal/mol), *R* is a gas constant (1.99 *·* 10^−3^ kcal/mol*·*K), and *T* is a temperature (310 K).

 We used all the available sequences of phosphofructokinases from the Ensembl data base [28]. If there are different directions of symmetric mutational pressure in sequences from the same alignment, evolutionary distances between them usually become higher [29]. To avoid that problem, we excluded those sequences in which GC-content in third codon positions (3GC) is lower than 50% [11]. Moreover, we excluded partial sequences (except those from Lamprey genome) and sequences with regions which cannot be aligned properly. Finally, we excluded sequences from different species of fish because there are several copies of tissue-specific phosphofructokinases in their genomes.

 Lancelet (*Branchiostoma floridae*) homologue of phosphofructokinases (XM_002607302) from vertebrates has been found with the help of BLAST search (http://blast.ncbi.nlm.nih.gov/Blast.cgi).

 Sequences have been aligned by MUSCLE algorithm included into MEGA 5 program [30]. We calculated JTT amino acid evolutionary distances between sequences [31]. The dendrogram has been built by UPGMA method [31].

## 3. Results and Discussion

 Activities of both muscle and liver phosphofructokinases were inhibited by average and high bolus doses of ethanol (see Table 1). Activity of muscle phosphofructokinase has been decreased 1.23 times after the admission of 2.5 g of ethanol per 1 kg of body weight and 1.48 times after the admission of 5.0 g per kg. Activity of liver phosphofructokinase has been decreased about 1.43 times after the admission of both average and high doses of ethanol (see Table 1).

 It was shown that alcohol consumption (both acute and chronic) leads to the decrease of ATP level and increase of ADP level in cells [32, 33]. So, the ATP/ADP ratio becomes lower during the period of ethanol intake. Elevated levels of ADP should have a stimulatory effect on the activity of phosphofructokinase [2]. However, our data showed that activities of muscle and liver phosphofructokinases can be somehow decreased by ethanol. This decrease of enzymatic activities is possible in case when the compensatory mechanism linked with ADP level increase is not working yet (during acute alcohol intoxication).

 One of the possible pathways of phosphofructokinase activity inhibition during acute alcohol intoxication may be linked with elevated NADH/NAD^+^ ratio. Ethanol consumption leads to the NADH accumulation (alcohol dehydrogenases and aldehyde dehydrogenases produce NADH from NAD^+^) [1, 34]. Isocitrate dehydrogenase is inhibited by elevated HADH level, allowing an increase in citrate level [1]. Citrate is a known inhibitor of phosphofructokinase activity [3, 34].

 As we have shown previously, acute alcohol intoxication (5 g per kg of body weight) causes decrease of muscle and liver pyruvate kinases activities (they become 1.47 and 1.35 times lower, resp.) [11]. Because of this reason, phosphoenolpyruvate level should increase during acute alcohol intoxication. Phosphoenolpyruvate is a known inhibitor of phosphofructokinase activity [3]. The most interesting fact about phosphofructokinase activity inhibition by citrate and phosphoenolpyruvate is that those substances require sufficient level of ATP for their action [3]. In other words, both citrate and phosphoenolpyruvate can inhibit phosphofructokinase activity when the level of ATP is relatively high [3]. Those conditions may be established during acute alcohol intoxication.

The nature of the inhibitory effect of ethanol on muscle and liver phosphofructokinases activities observed in *in vivo* experiments has been clarified in the *in vitro* experiment (see Table 2). Activity of muscle phosphofructokinase was inhibited by the 500 mM ethanol only. Such a high concentration of ethanol is impossible inside living cells. It means that even though ethanol can directly interact with muscle phosphofructokinase and inhibit its activity, that kind of direct inhibition is possible only *in vitro*.

 Activity of liver phosphofructokinase has been significantly inhibited by 100 mM ethanol (see Table 2). That concentration of ethanol is very high, and it may be established in living cells after the enormous alcohol intake (100 mM is equal to 4.6‰). It is known that blood alcohol levels higher than 5‰  are lethal. However, there are some known cases when blood alcohol levels were around 8–10‰ [35]. So, direct inhibition of liver phosphofructokinase activity is possible in humans after the intake of very high doses of alcohol.

 Peak blood alcohol level in rats after the single intake of 5.0 g per kg of ethanol may reach 108.0 ± 2.3 mM [17]. So, inhibitory effect of acute alcohol intake can be direct. It means that inhibition of liver phosphofructokinase activity after the intake of high dose of alcohol may happen because of both direct and indirect mechanisms (probably, due to inhibition by citrate and phosphoenolpyruvate in the presence of sufficient ATP concentration).

 ADP is an allosteric activator of phosphofructokinase (PFK) [2]. The site for ADP binding includes Asp173, Met174, Asp179, Tyr214, Phe308, Asn341, Ser377, Asn381, Phe538, Asp543, and Phe671 residues [2]. According to the results of molecular docking of ADP to models of rat and human muscle and liver phosphofructokinases, all these eleven amino acid residues are predicted as those binding ADP. Phenylalanine residues (Phe308, Phe538, and Phe671) can participate in either cation-pi or hydrophobic interactions with adenine. Asp543 is able to make a hydrogen bond with –NH_2_ group of adenine.

**Figure 1:**
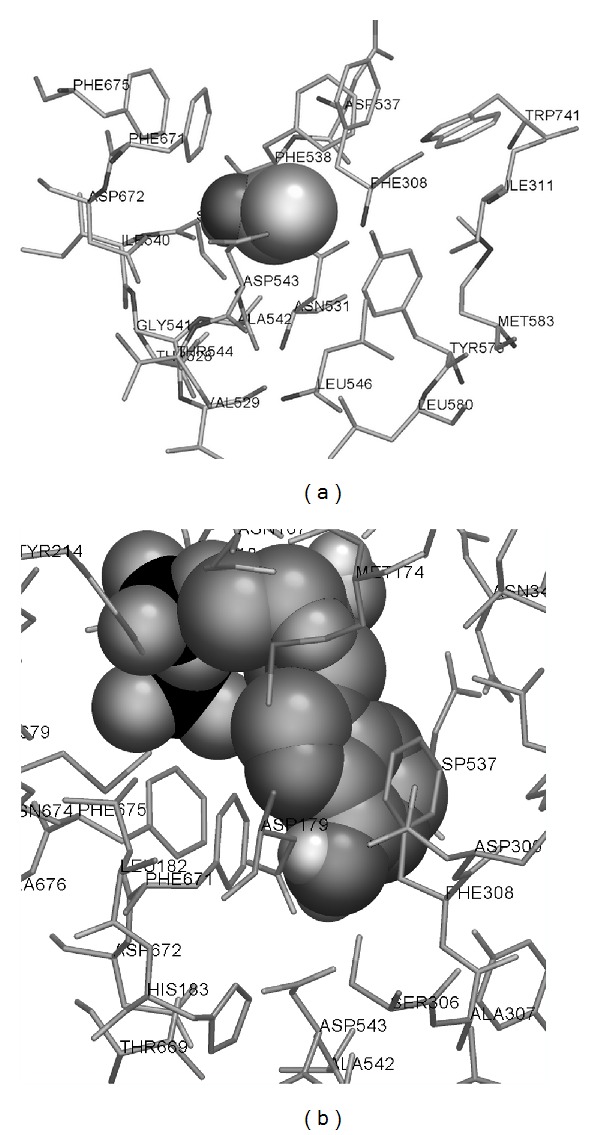
Results of the docking of ethanol (a) and ADP (b) to the model of rat muscle phosphofructokinase. Ligands are shown by spheres, interacting amino acid residues are shown by cylinders, and other amino acid residues of the protein are hidden.

As one can see in Figure 1, ethanol may be bound by the same part of ADP binding site which is able to bind adenine, at least, in the model of rat muscle phosphofructokinase. Three phenylalanine residues (Phe308, Phe538, and Phe671) are able to participate in either hydrophobic or cation-pi interactions with ethanol, while Asp543 usually takes part in polar interactions.

**Figure 2:**
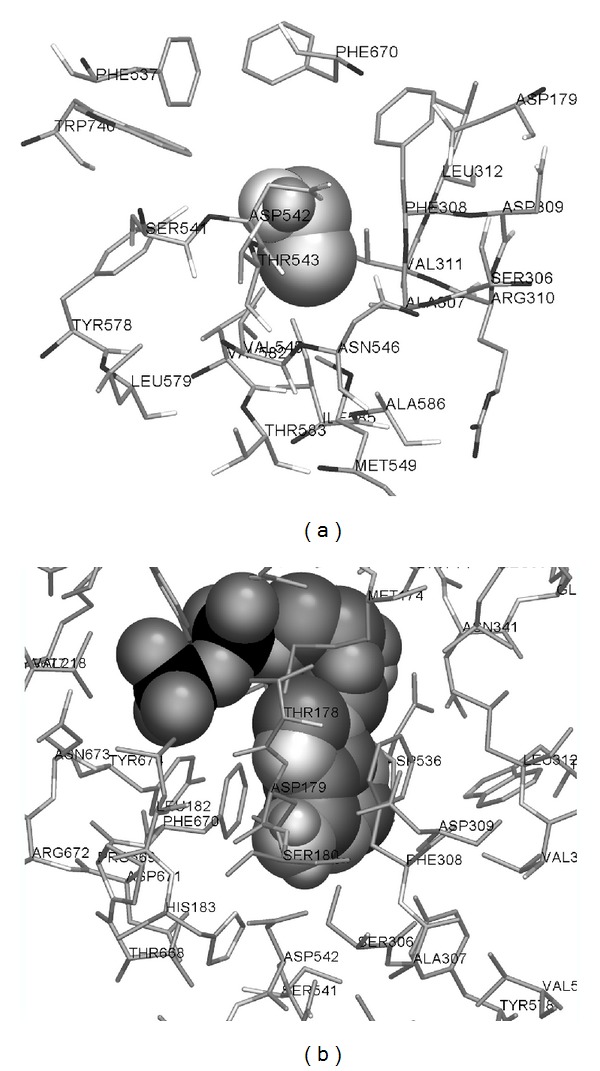
Results of the docking of ethanol (a) and ADP (b) to the model of rat liver phosphofructokinase. Ligands are shown by spheres, interacting amino acid residues are shown by cylinders, and other amino acid residues of the protein are hidden.

In the same manner, ethanol can be docked to the adenine binding pocket of ADP binding site from the model of rat liver phosphofructokinase (see Figure 2). Even though positions of ethanol in the allosteric site of both enzymes are similar, there are some differences. In the variant of ethanol docking to the rat muscle phosphofructokinase with lowest total free energy of binding, the main role is played by Phe538 (its decomposed free energy of binding is equal to −0.4589 kcal/mol) and Asp543 (−0.4915 kcal/mol). In the best docking of ethanol to the rat liver phosphofructokinase, the main role is played by Val545 (−0.3043 kcal/mol). Val545 participates in hydrophobic interactions with ethanol.

**Figure 3:**
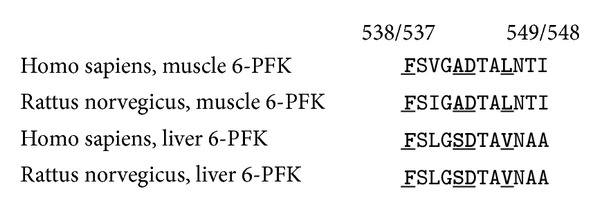
Alignment of the linear part of the predicted ethanol-binding site for muscle (amino acids 538–549) and liver (amino acids 537–548) rat and human phosphofructokinases. Amino acid residues interacting with ethanol according to the results of molecular docking are shown by bold underlined type.

As one can see in Figure 3, Val545 from liver phosphofructokinase is aligned with Leu546 from muscle phosphofructokinase. Val545 seems to be more suitable for hydrophobic interactions with ethanol than Leu546. As to Ala542 from muscle phosphofructokinase, it has a positive free energy of binding with ethanol. The place of Ala542 is occupied by Ser541 in liver phosphofructokinase. So, some amino acid substitutions in the linear part of predicted ethanol binding site might influence its ability to bind that ligand (see Figure 3).

It is interesting to mention that the known ethanol-binding site of the LUSH protein (according to 1OOF [9], 3B7A, and 3B86 [10] 3D structures) also includes hydrophobic amino acids interacting with –CH_2_–CH_3_ tail of ethanol (Val58 and Phe113). However, hydrophilic part of the ethanol molecule (–OH group) makes hydrogen bonds with Ser52 and Thr57 residues of LUSH [9, 10], while in case of phosphofructokinases, that group participates in cation-pi and polar interactions.

Average free energies of binding for ATP, ADP, and ethanol are given in Table 3. It is important to highlight that free energies of binding for ADP are lower than those for ATP. As one can see in Table 4, the difference in binding constants for ADP and ATP is rather high. So, the site for ADP binding should be very specific [2].

Average free energies of binding for ethanol are about 3.3 times higher than those for ADP if we are talking about muscle phosphofructokinases. For liver phosphofructokinases, average free energies of binding for ADP are about 3.0 times lower than those for ethanol. Liver enzymes bind ethanol better than muscle enzymes, while those liver enzymes bind ADP worse than muscle enzymes (see Tables 3 and 4).

Of course, binding constants for ethanol are much lower than those constants for ADP. It means that ethanol may somehow bind allosteric site for ADP but only at rather high concentrations. Indeed, results of *in vitro* experiment support the data from Table 4: concentration of ethanol should be 100 mM and higher for liver and 500 mM and higher for muscle phosphofructokinase.

On the other hand, average free energy of ethanol binding for LUSH protein is −2.03 kcal/mol, which is just 8.5% lower than average free energy of binding for the model of human liver phosphofructokinase (9.6% lower than that of the model of the rat enzyme). The constant of ethanol binding for LUSH protein (26.86) is just 1.29 times higher than that for the model of human liver phosphofructokinase (1.33 times higher than for the model of rat liver phosphofructokinase). Interestingly, according to the Docking Server results for LUSH protein and ethanol, decomposed free energy of hydrogen bond formation by Ser52 (−0.1157 kcal/mol) is lower than the decomposed free energies of hydrophobic interactions with Phe113 (−0.4007 kcal/mol) and Val58 (−0.2221 kcal/mol), while Thr57 is mentioned among amino acids participating in other interactions (−0.0404 kcal/mol). That data approves a high probability of ethanol binding by the nonspecific site of liver phosphofructokinase described in this study, since Δ*G* and *K* for the specific binding site of LUSH are close to those for ADP-binding pocket of modeled enzymes.

**Figure 4:**
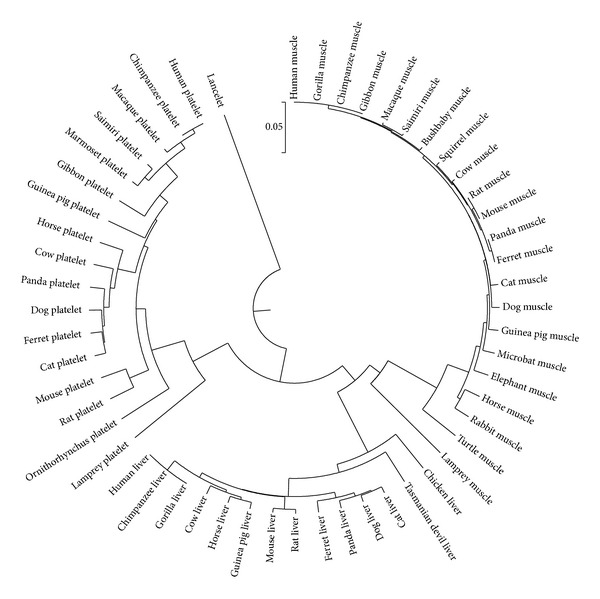
A circle dendrogram representing phylogenetic relations between muscle, liver, and platelet phosphofructokinases of vertebrates. Homologous protein of the lancelet is used as an outgroup. Phylogenetic tree is built by UPGMA method based on the JTT amino acid evolutionary distances.

In our opinion, liver phosphofructokinases are directly inhibited by lower concentrations of ethanol than muscle phosphofructokinases because of certain amino acid substitutions fixed in the linear part of its binding site. Divergence of muscle and liver phosphofructokinases happened in the period between 713.2 and 535.7 Millions of years ago (Mya), just like the divergence between muscle and liver pyruvate kinases [11]. As one can see in Figure 4, a protein from lancelet proteome that is homologous to phosphofructokinases of vertebrates is an outgroup. The time of the divergence between lancelets and the predecessor of vertebrates is about 713.2 Mya [36]. There are tissue-specific (muscle and platelet) phosphofructokinases in the proteome of lamprey (see Figure 4). It means that divergence between three types of phosphofructokinases happened before the divergence of lampreys and other vertebrates (before 535.7 Mya [36]). According to the dendrogram from Figure 4, muscle and liver phosphofructokinases show more similarity with each other than each of them with their homologue expressed in platelets.

## 4. Conclusions

Activity of liver phosphofructokinase can be inhibited by high doses of ethanol (5 g per kg of body weight resulted in 100 mM and higher peak blood levels) due to the binding of ethanol by the allosteric regulatory site.

Activity of muscle phosphofructokinase can also be inhibited due to the binding of ethanol at a similar site, while the concentration of that ligand necessary for enzyme inhibition (500 mM) is higher than any possible ethanol concentration in living cells.

Inhibition of muscle and liver phosphofructokinases activities by a single intake of average dose of alcohol (2.5 g per kg of body weight) is indirect and it is probably associated with increased NADH/NAD^+^ ratio.

## Figures and Tables

**Table 1:** Activities (nmol/mg/min) of muscle and liver phosphofructokinases after the acute alcohol intoxication.

Enzymes	Control group (*n* = 7)	Ethanol dosage		
		1.0 g/kg (*n* = 7)	2.5 g/kg (*n* = 7)	5.0 g/kg (*n* = 8)
Muscle PFK	97.9 ± 13.2	94.7 ± 7.0	79.5 ± 9.4*	66.3 ± 7.1*
Liver PFK	8.4 ± 0.7	8.6 ± 0.8	5.9 ± 0.5*	5.8 ± 0.5*

Significant (*P* < 0.05) differences with control group are shown by asterisks (*).

**Table 2:** Activities (nmol/mg/min) of phosphofructokinases in muscle and liver supernatants after the addition of ethanol.

Enzymes	Control (*n* = 6)	Concentration of ethanol			
		5 mM (*n* = 6)	50 mM (*n* = 6)	100 mM (*n* = 6)	500 mM (*n* = 6)
Muscle PFK	90.3 ± 7.9	75.2 ± 14.7	85.9 ± 7.2	82.4 ± 10.2	67.3 ± 6.6*
Liver PFK	8.6 ± 0.6	8.2 ± 0.2	8.1 ± 0.7	5.3 ± 0.7*	5.0 ± 0.6*

Significant (*P* < 0.05) differences with control group are shown by asterisks (*).

**Table 3:** Average free energies of binding for ADP, ATP, and ethanol with human and rat muscle and liver phosphofructokinases.

Ligand	Enzyme			
	Rat muscle	Human muscle	Rat liver	Human liver
ADP	−6.011 kcal/mol	−6.016 kcal/mol	−5.724 kcal/mol	−5.637 kcal/mol
ATP	−5.375 kcal/mol	−4.413 kcal/mol	−5.255 kcal/mol	−3.623 kcal/mol
Ethanol	−1.812 kcal/mol	−1.821 kcal/mol	−1.852 kcal/mol	−1.871 kcal/mol

**Table 4:** Binding constants for ADP, ATP, and ethanol with human and rat muscle and liver phosphofructokinases.

Ligand	Enzyme			
	Rat muscle	Human muscle	Rat liver	Human liver
ADP	17049.58	17188.33	10706.98	9298.64
ATP	6080.97	1278.59	5006.03	355.28
Ethanol	18.86	19.14	20.13	20.76
